# Psychiatric Mobilities: Palestinian Patients at the Lebanon Hospital for Mental Diseases, 1900–1948

**DOI:** 10.1093/shm/hkag071

**Published:** 2026-08-14

**Authors:** Chris Sandal-Wilson

**Keywords:** psychiatry, migration, borders, colonialism, Palestine, Lebanon, Middle East

## Abstract

Between 1900 and 1948, hundreds of patients from Palestine travelled north to the Lebanon Hospital for Mental Diseases outside Beirut for treatment, a pattern of psychiatric mobility that endured the collapse of the Ottoman empire and the colonial partition of the Levant by the British and French. Drawing on the hospital’s records and the archives of the British mandate government of Palestine, this article examines why so many Palestinians travelled for treatment, and how this pattern of mobility was rearticulated in the wake of war, anti-colonial revolt, and colonial repression. As well as underlining the need to escape methodological nationalism in the history of psychiatry and to track continuities across conventional chronological boundaries, this case study emphasises the agency of families of patients as they navigated new borders and the seismic social and political developments of these pivotal decades in the making of the modern Middle East.

## Introduction

In August 1900, the Lebanon Hospital for the Insane—as it was initially known—opened its doors to patients for the first time. Established on the foothills of Mount Lebanon a few kilometres to the south-east of the bustling port city and Ottoman provincial capital of Beirut, the hospital was the result of a vigorous fundraising campaign throughout Europe and the United States by a sexagenarian Swiss Quaker missionary, Theophilus Waldmeier, and his wife, Fareedy Saleem.^1^ By March 1901, over fifty patients had been admitted to the hospital, and many more brought to the medical superintendent for examination.^2^ Nearly half of this initial intake came from Mount Lebanon itself; indeed, it was deemed noteworthy that one ‘very excited patient was brought, bound with ropes on a donkey’s back, from a village three days’ journey’ away.^3^ But in among the first patients admitted to the hospital were three who had travelled much further still, journeying over a hundred miles north from Ottoman Palestine. In August 1901, a Muslim merchant from Nablus was admitted to the hospital; in December that same year, two more patients, a Jewish gardener and a Greek Catholic blacksmith, arrived at the hospital, travelling up from Jaffa.

They were the first of many patients from Palestine to be admitted to the Lebanon Hospital for Mental Diseases or ʿAsfuriyyeh—as the hospital was also known—across the first half of the twentieth century.^4^ Between the hospital’s opening in 1900, and the *nakba* or catastrophe of Palestinian displacement and dispossession in 1948, well over three hundred Palestinians travelled north to Beirut for psychiatric treatment.^5^ In many respects, this pattern of psychiatric mobility is unsurprising. Prior to the First World War travel within the Levant was, after all, internal to the Ottoman empire and quite routine. More than this, there is evidence that at least some Palestinian patients were travelling even greater distances—to Cairo and to Istanbul, among other destinations—for psychiatric treatment in these years.^6^ But if Levantine psychiatric mobilities appear self-explanatory for the late Ottoman period, their persistence in the tumultuous decades following the First World War, the collapse of the Ottoman empire, and the partition of the region between the British and French would seem to require more by way of explanation—especially as psychiatric provision *within* the newly drawn borders of mandate Palestine grew significantly across these years, too.^7^

Drawing on the hospital’s records and the archives of the British mandate government of Palestine, this article examines why Palestinians travelled for psychiatric treatment, and how this pattern of mobility was rearticulated in the wake of war, anti-colonial revolt, and colonial repression. While anthropologists have often discussed transnational therapeutic itineraries in the present as a response to the dismantling of national healthcare systems under neoliberalism, sanctions, or war, focusing on the Levant in the first half of the twentieth century yields a different perspective.^8^ Local histories of violence certainly mattered in this context, though their impact on psychiatric mobilities was far from straightforward: if the First World War interrupted the flow of patients north, the Palestinian great revolt and brutal British counterinsurgency between 1936 and 1939 was accompanied by a spike in Palestinian admissions to ʿAsfuriyyeh. But rather than something exceptional, always necessitating an explanation of the same order of magnitude as war, travelling for treatment in this context emerges as a phenomenon driven by a mix of often quotidian considerations: from concerns about scarcity and safety, to the desire for a particular standard of care or for unwell relatives to be at a certain degree of proximity (or, conversely, distance). In their ordinariness, then, the itineraries of psychiatric patients examined here underline the artificiality of fettering histories of psychiatry in the region to the borders which emerged after the First World War—a framing that naturalises borders and denaturalises mobility, inverting their order of appearance. After all, as Toufoul Abou-Hodeib reminds us: ‘transnational movement was regional movement even before it became transnational’.^9^

If this pattern of travelling for treatment makes clear the need to escape methodological nationalism in the history of psychiatry, tracking psychiatric mobilities across the first half of the twentieth century—and in particular across the transition between Ottoman rule and the mandate system imposed on the region in the aftermath of the First World War—suggests the importance of thinking across conventional chronological boundaries, too.^10^ Rather than an immediate or total rupture, the transition from empire to mandates was marked by significant continuities, with the flow of patients from Palestine to ʿAsfuriyyeh resuming shortly after the end of the war. If this is broadly in line with what historians of the mandate period increasingly argue,^11^ focussing on mobile subjects has proven a particularly productive way to uncover continuities: characters such as the muleteer, pilgrim, smuggler, and merchant whose cross-border movements kept alive an alternative regional geography even as mandate states emerged and solidified.^12^ Tracking psychiatric mobilities further de-centres colonialism as the only or even most important point of reference for Levantines in the interwar decades. Across this period, ʿAsfuriyyeh offered an option to Palestinians which was beyond the borders of the newly created British mandate, and—as Joelle Abi-Rached has shown, in her *longue durée* history of the hospital from its inception to its closure during the Lebanese civil war in 1982—represented at least as much a local as an international, colonial, or missionary institution.^13^

Following the case of Palestinian patients at the Lebanon Hospital for Mental Diseases across the first half of the twentieth century, this article proceeds in three parts. Starting in the late Ottoman period, it establishes who these patients were and why they looked to Beirut for treatment. It then moves into the mandate era, showing how this pattern of psychiatric mobility resumed following the First World War and adapted to the major political and social changes of the interwar years. The final section focuses in closely on the last decade of mandate rule, when anti-colonial revolt, colonial counterinsurgency, and the Second World War all impacted on these psychiatric mobilities in complex ways. Across all three stages, this article emphasises the role played by families in shaping and sustaining these transnational therapeutic itineraries.^14^ If historians increasingly recognise the agency of families within the micropolitics of psychiatric care,^15^ the case of Palestinian patients at ʿAsfuriyyeh extends this; it brings into view how families might engage not only in complex negotiations with medical experts and government officials around mental illness and its treatment, but do so across borders, navigating the unstable political tectonics of the region with no small degree of virtuosity. The agency of often well-to-do families in this arena exceeds any simplistic framing as collaboration or resistance in relation to colonial power; here again, colonial rule appears as one of a number of points of reference, not the sole determinant of behaviour. Power dynamics and asymmetries between family, colonial state, and psychiatric institution mattered. But so too did those dynamics and asymmetries that existed within the family, between psychiatric patients and the relatives who sought relief for and sometimes from them—though recovering these intra-familial dynamics takes us to the very limits of the archive.

## Travelling for treatment in the late Ottoman Levant

The three patients admitted to the Lebanon Hospital for Mental Diseases from Palestine in 1901 were but the first of many to travel north to Beirut for treatment in the final years of Ottoman rule. Before 1918, over 150 patients from Palestine were treated at ʿAsfuriyyeh.^16^ Palestinians consistently made up a relatively significant proportion of admissions: in 1908, for instance, 18 out of a total of 116 patients admitted were from Palestine.^17^ In other contexts, these figures might have raised alarm. In one of the rare works to consider psychiatric mobilities in a comparable regional context, Matthew Heaton argued that for authorities at the Accra lunatic asylum in colonial Gold Coast, patients from elsewhere in West Africa represented a problem population, and their discharge and deportation was mooted as a possible solution to the institution’s overcrowding in the early twentieth century.^18^ Palestinians at ʿAsfuriyyeh were viewed quite differently; indeed, as Ottoman subjects who crossed no borders to travel north for treatment in this period they were hardly viewed as a distinct patient population at all, but were disaggregated in the hospital records and counted only by town of residence.^19^ Far from a burden, their presence was cause for celebration, evident in the hospital’s annual reports. In 1906, for instance, the medical officer Dr Harry Thwaites boasted of the hospital’s regional reach, reporting proudly ‘how wide has been our sphere of usefulness, patients having been drawn from various parts of Syria and Palestine, as well as from Armenia, Arabia, and Egypt’.^20^ If hospital administrators lauded such admissions patterns as reflecting the hospital’s growing reputation, there is a more prosaic reason that this was cause for celebration: it widened the pool of potential paying patients whose fees could supplement donations and enable the hospital to continue to offer free treatment to the poorest.^21^

Who were these patients from Palestine, and why did so many make the journey to Beirut for treatment in the years before the end of Ottoman rule? In many ways the initial intake of three patients in 1901 offers a good starting point for understanding the demographics of the hospital’s Palestinian patient population. Like the vast majority of patients they were young, aged between nineteen and thirty; two came from Jaffa, one of the most populous towns in Palestine, and the third from Nablus, towards the north and indeed part of the same Ottoman administrative district as Beirut; they had a range of professions, including the single most common profession recorded for Palestinians in the hospital across this period, merchant; they were drawn from Palestine’s Muslim, Christian, and Jewish populations; and they were all given a diagnosis of dementia praecox, which was applied when symptoms such as delusions and disorganisation of thought, speech, and behaviour presented in younger people, and would be superseded by schizophrenia in the interwar years.^22^ This diagnostic profile was certainly evident in the one patient from this group whose case notes survive: Ibrahim H., the Jewish gardener admitted from Jaffa in December 1901, was reported to have ‘ideas of grandeur’, and claimed that ‘he was the son of Rothschild and called himself Charlie Rothschild’, a reference to the wealthy Baron Rothschild who underwrote struggling European Jewish settlements in Palestine in the last decade of the nineteenth century;^23^ as late as 1910 Ibrahim was described as ‘[s]till deluded that he has plenty of money’.^24^ And while two-fifths of patients from Palestine before 1918 were women, this group of three male patients also captures the slight preponderance of male over female patients at ʿAsfuriyyeh across the hospital’s lifetime—a phenomenon which has been linked to patriarchal social dynamics that prioritised the well-being of male breadwinners and shied away from entrusting female relatives to foreign doctors.^25^

Each of these points deserves elaboration, but the range of religious backgrounds recorded for Palestinian patients might stand out as particularly striking. The Lebanon Hospital for Mental Diseases, after all, had been founded by a Quaker missionary, with a non-denominational Christian service of worship held on site each Sunday since its foundation. Yet from the start, the hospital took pains to emphasise its openness to all: the very first intake of just fifteen patients in August 1900 included, the hospital reported proudly, ‘Jews, Mohammedans, Druses, Greeks, Maronites and Protestants, so that nearly all the Holy Land religions are represented’.^26^ In 1906, the medical officer returned to this theme, celebrating ‘the inclusion amongst the patients of men and women belonging to all creeds and religions’ as ‘gratifying proof to all that [the hospital] is being conducted in the interests of the people, as an international institution, and quite irrespective of denomination’.^27^

There was nothing particularly unusual in Muslim and Jewish patients accessing a Christian mission institution. This dynamic had been playing out in both Lebanon and Palestine since at least the mid-nineteenth century, not only in the field of health but also education; the failure of Christian missionaries to convert Muslims and Jews in any significant numbers had led them to shift their focus from direct methods of proselytisation to indirect methods of reaching people through education and health instead.^28^ But when set against the backdrop of a century more often viewed through the lens of Arab-Jewish separation and conflict, the number of Jewish patients is arresting, comprising just under half of admissions to ʿAsfuriyyeh from Palestine before 1918. That so many Jewish patients made the same journeys north to seek out and receive treatment at the same hospital as their Muslim and Christian neighbours is a reminder of the fallacy of assuming separation or conflict for the late Ottoman period. Historians have for decades argued for the value of a ‘relational’ approach that attends to Arab-Jewish interaction; this is as true of the history of psychiatry as it is for other, better-studied areas of social and economic life.^29^

Having established some sense of who these patients were, why did so many Palestinians look north to ʿAsfuriyyeh in the late Ottoman period? Was it simply a result of the absence of alternatives? At one level, it is true that ʿAsfuriyyeh had no real rival within Palestine in these years. But the picture with regards to provision in Palestine is more complex than might be assumed, and other considerations, such as the quality and proximity of care, were factors, too. Alongside a range of non-medical possibilities—from shrines reputed to have healing powers to shaykhs able to exorcise malignant spirits—there were certainly some medicalised options available within Palestine, including a hospice at a Jerusalem convent and a number of general hospitals in major towns.^30^ And for Jewish patients, the picture was more complicated still. In 1895, a Jewish women’s charitable organisation had established the Ezrath Nashim Home for the Insane and Incurable in Jerusalem, in response to the needs of the growing number of European Jews arriving in Ottoman Palestine. Initially just a single room in the Old City, the Ezrath Nashim home expanded across the last decades of Ottoman rule, moving to a new extramural site and accommodating nearly fifty patients by the end of the First World War.^31^

While the Ezrath Nashim home did not become a specialist psychiatric institution until the 1920s, it is insufficient to simply dismiss it entirely: its shortcomings were more specific, and so reveal something of the motives of the families of patients who chose to go elsewhere. At a very basic level, and remembering the skew towards male admissions from Palestine at ʿAsfuriyyeh, it almost exclusively admitted women, before a second building for male patients was added in 1910.^32^ And prior to the 1910s, the home also consistently admitted more ‘incurable’ than ‘insane’ cases. In 1901, for instance, the same year the first three patients from Palestine made their way north to ʿAsfuriyyeh, the home admitted three ‘insane’ patients of their own—and fifteen ‘incurable’ patients.^33^ This emphasis on ‘incurable’ cases is compounded by the fact that the Ezrath Nashim home only hired a specialist in mental diseases in 1920.^34^ As we have seen, most of the patients who found their way to ʿAsfuriyyeh were young: almost two-thirds of patients were under the age of thirty on admission. It is not hard to imagine that the atmosphere of therapeutic pessimism attendant on the skew in the Ezrath Nashim’s population towards ‘incurable’, ageing cases, and the absence of any psychiatric specialist, would have led families to look elsewhere, even had beds been readily available. Rather than see the decision to travel to the Lebanon Hospital for Mental Diseases as necessitated by the absolute absence of alternatives, then, the quality and nature of the care available appear to have been important considerations—suggesting the success of the hospital’s efforts ‘not to be a place of last resort’, as Joelle Abi-Rached has put it.^35^

If a lack of direct alternatives was one factor, and consideration of the quality and nature of care another, it is also worth noting the simple proximity of the Lebanon Hospital for Mental Diseases for many patients. A third of patients admitted before 1918 came from Jerusalem, with Jaffa the second most common place of residence recorded.^36^ These were the two most populous urban centres and their prominence is unsurprising. But after Jerusalem and Jaffa it is striking that northern towns were heavily represented, towns such as Haifa, Nablus, Nazareth, Acre, Safad, and Tiberias. These towns were not only physically closer to Beirut, but were in fact part of the same Ottoman administrative unit, and caught up in the city’s gravitational pull: litigants, merchants, and students from the north of Palestine flocked to the thriving port city of Beirut in this period.^37^ The families of psychiatric patients appear to have followed suit.

The scarcity, quality, and proximity of psychiatric care emerge as important factors in the hospital’s annual reports and admissions lists. But a close reading of the one surviving patient case book in the archive enables deeper insight into individual stories and so a more richly textured sense of psychiatric mobilities in this period. While only one patient case book—for male patients admitted between 1904 and 1909—is extant, this can nevertheless be contextualised to uncover additional, key elements in this story: above all, the significance of return migration and the expansive role played by the families and friends of patients.

To take migration first, this was, as Joelle Abi-Rached notes, one of the most important elements in psychiatric thinking at the Lebanon Hospital for Mental Diseases almost from its foundation.^38^ This is unsurprising: migration was perhaps the defining feature of life in late Ottoman Lebanon. From the late 1880s onwards, hundreds and later thousands left Lebanon each year for the Americas; by the outbreak of the First World War, it is estimated almost a third of the population of Mount Lebanon had emigrated.^39^ The medical authorities at ʿAsfuriyyeh had to get to grips with this phenomenon quickly. In 1901, Dr Otto Wolff, first medical superintendent of the hospital, noted how ‘[e]migration to America and other places is too often the cause of mental disorder’. He explained: ‘I often hear the history of having gone to America, of bringing back a sum of money which is a small fortune in Syria, then of being robbed of it, and the sorrow brought on thereby causing loss of reason.’ Those who were already rich, he continued, had a different problem: ‘Many of them visit places abroad, where they lose their money by gambling, and where they lead bad lives; they often come back quite insane’.^40^ If Wolff’s explanation was anecdotal in nature, focussed on sudden shocks and traumatic reversals in fortune, in 1907, his successor Dr Thwaites offered another interpretation, centred on heredity and degeneration. Return migrants were disproportionately represented not because of mere individual misfortune, but because migration alchemised inherited weakness into overt insanity. Thwaites reasoned that ‘it is probable that many, in whom mental unsoundness is latent, might have escaped but for the additional strain to which they exposed themselves in countries where life is more complex, and problems of existence more involved’.^41^ In this way, Wolff and Thwaites were representative of two strands in psychiatric thinking on migration which stretched back to the late nineteenth century: one which attributed mental illness directly to the stresses of migration, and another which held that migration simply brought to the surface the inherent susceptibility of particular groups to mental disease.^42^

Wolff and Thwaites did not disaggregate the patient population, but the surviving case book makes clear there were Palestinian return migrants at the hospital. While never quite reaching the same proportions as emigration from Mount Lebanon, tens of thousands of Palestinians left for the Americas in the decades before the First World War.^43^ The popular image that has tended to dominate the representation of return migrants, at the time and since, has been that of the successful, decidedly masculine figure whose newfound wealth was reinvested in extravagant mansions in the small towns of Palestine, ‘rupturing… [the] parochial, more rural past’ in a highly visible way.^44^ Those Palestinian return migrants whose stories find their way to us through the archives of the Lebanon Hospital of Mental Diseases offer something of an inversion of this image, highlighting the tensions and anxieties migration might unleash, not just the profits and upward mobility it could unlock.

Yakub Z. was one of half-dozen or so Palestinian patients in the surviving case book whose lives reveal how migration and mental illness could be entangled. Admitted to ʿAsfuriyyeh in December 1907, at the age of twenty, Yakub’s symptoms of emotional dullness, disinterest in his surroundings, and apparently random laughter, led to him being given a diagnosis of dementia praecox. His personal history seemed to hold the key to understanding his condition. He had been a shopkeeper from Bethlehem and, like so many of his contemporaries, had left for America over 3 years earlier, when he would have been just seventeen.^45^ After a year there he appeared to develop a mental disease which manifested itself in ‘careless laughing and smiling and severe mental incapacity’, as his notes put it, and he returned to his family back in Bethlehem. He remained with them, apparently unchanged, for over 2 years, before his father finally brought him to ʿAsfuriyyeh. In January 1908, however, just a month into his stay at the hospital, he was discharged back to his father, with no improvement recorded.^46^

Yakub’s is but the most abbreviated of a story which finds repetition in other case files, of a young man travelling to America, suffering some kind of mental breakdown, and returning for treatment.^47^ These stories highlight how histories of international migration to and from the Americas could link up to regional patterns of medical migration, as Palestinian return migrants judged to suffer from mental illness found themselves on the move once again, this time for treatment at ʿAsfuriyyeh. But Yakub’s story also directs attention to a second important element in this picture: the role of families. As recent histories of psychiatry in general and of ʿAsfuriyyeh in particular make clear, families were often key actors in the admission, discharge, and indeed institutional experiences of their relatives.^48^ Yakub’s father was recorded as bringing him to the hospital initially, and receiving him when he was discharged. He was far from unique. Izzat S., for instance, a Muslim clerk from Jerusalem who was admitted in October 1908 at the age of nineteen with a diagnosis of melancholia, was brought to the hospital by his mother and father;^49^ while Samuel L., a Jewish engineer living in Jaffa and admitted to the hospital in October 1907 with a diagnosis of alcoholic delirium, was accompanied by his wife, who later reportedly ‘insists on receiving him and thinks him cured’.^50^ In some cases, friends rather than family played this role. This was true of Ibrahim S., a Greek Catholic man from Jaffa who was admitted in November 1907. It was his friends who ‘insisted on his removal’, just a month later.^51^

Case notes also reveal that families could continue to be involved in the management of mentally ill relatives while they remained patients at ʿAsfuriyyeh. Far from being cut off from the outside world, patients were frequently recorded as receiving visits from family and friends. Indeed, these visits seem to have been so routine that those that warranted recording in patient case notes were in some way exceptional, usually because they were taken to offer insight into the patient’s mental condition. Jules G. is a case in point. A Protestant merchant from Jaffa, Jules was brought to the hospital in June 1909. His mother was recorded as visiting him a few months later, in September. But she was one of the main subjects of his delusions: he had told hospital staff that she was a prostitute and ‘that he shot at her four times and regrets that he did not shoot straight’. When she visited, he insulted her and ‘flew into a temper’. She nonetheless returned in April 1910 and asked for her son to be given a trial period at home to see how the family could manage. Jules was discharged into her care—but he returned to the hospital in March 1912, and remained there until his death from pneumonia in November 1917.^52^

Taken together, admissions statistics and patient case notes reveal much about how and why Palestinians came to seek treatment at the Lebanon Hospital for Mental Diseases in the last years of Ottoman rule. To the usual cast of characters in histories of mobility, they add striking new figures: the young Jewish patient whose family baulked at the idea of condemning a relative to join the ranks of ‘incurables’ at the Ezrath Nashim home and whisked them north instead; the Palestinian return migrant whose journey did not come to an end once home but continued onwards, as they were set in motion yet again by their illness; the family of the psychiatric patient, whose journeys to and from ʿAsfuriyyeh became so regular that hospital staff only took note when these appeared to unmask a patient’s progress, or lack thereof. As a set, these examples stretch how we might think of psychiatric mobilities. Rather than any one-time journey to and—assuming they were ever discharged—from the hospital suggested by annual reports and their deceptively straightforward breakdowns of patient demographics by place of residence, the kinds of psychiatric mobilities which emerge through close reading of patient case notes are kaleidoscopic in directionality and duration, linking scales of movement and iterating across weeks, months, and years. What happened to this complex world of psychiatric mobilities, which Palestinian patients at ʿAsfuriyyeh and their families had knitted together in the twilight years of Ottoman rule, in the face of war, collapse, and the colonial partition of the Levant?

## Psychiatric mobilities between empires

The First World War posed an existential challenge to the Lebanon Hospital for Mental Diseases. Good relations with Ottoman authorities locally meant that the British medical officer and matron were permitted, exceptionally, to remain on site during the war, when they were technically enemy subjects.^53^ But the war plunged much of the Levant, and Mount Lebanon in particular, into misery, as conscription, famine, hyperinflation, and epidemic disease ravaged the population.^54^ Against this backdrop, admissions in general plummeted, with the number of patients travelling from further afield in particular collapsing. As Dr Henry Watson Smith, medical superintendent since 1909, explained in the middle of the war years, ‘the present increased difficulties of travelling and scarcity of money generally prevents people bringing patients to the hospital who can be managed at home’.^55^ This situation persisted into the final year of the war, with patient admissions falling further. Watson Smith read tragedy into these figures: ‘[m]eans of travelling are few and expensive and owing to the generally prevalent distress, no doubt many cases have died which under ordinary circumstances would have been brought to the hospital’.^56^

The British entered Jerusalem in December 1917, and the French landed in Beirut in October 1918, but the political settlement and indeed borders of the post-Ottoman Levant—with the League of Nations granting Britain the mandate for Palestine and France the mandate for Syria and Lebanon—took a number of years to reach their final shape. The border between Palestine and Lebanon was adjusted a number of times across the 1920s, and only officially approved and recognised by the League of Nations in the 1930s.^57^ Rather than a moment of irreversible rupture, then, the post-war partition of the Levant was a process, and an uneven one at that. While inhabitants of the immediate border regions could cross freely using border passes under the Bon Voisinage Agreement of 1926, others hoping to cross from Palestine into Lebanon had to hold valid travel documents with visas, issued by the French consul in Jerusalem.^58^

In spite of these upheavals, in 1920 the Lebanon Hospital for Mental Diseases was able to report that the pre-war pattern of patients travelling from across the region for treatment had revived.^59^ In 1924, the hospital’s annual report returned to a well-worn theme: the diversity of its patient population. While about half of patients were ‘natives of Syria’, it was reported, ‘the remainder are extraordinarily diverse in race and nationality’, coming ‘from all parts of the Near East, from Jerusalem, Nazareth, Damascus, from as far east as Baghdad, from Persia, Asia Minor, from the islands of Greece, Cyprus, Egypt, and places in North Africa’.^60^ This continued across the interwar decades: in 1935, the medical director took pride in reporting to the general committee in London ‘that inquiries for accommodation now lie on my table from Aleppo, Cyprus, Palestine, Transjordania, and Egypt’.^61^ ʿAsfuriyyeh admitted nearly forty patients from Palestine over the 1920s, and just under a hundred across the 1930s. As in the late Ottoman period, these numbers are not in absolute terms large: in an average year, just a handful of patients might be sent north for treatment. But this is in line with numbers from the pre-war period, suggesting the endurance of this pattern of psychiatric mobility across the transition from empire to mandates.

Historians increasingly emphasise continuities between the Ottomans and their British and French successors.^62^ But the persistence of this pattern nonetheless requires explanation, and not just because the colonial border between Palestine and Lebanon introduced new bureaucracy. Across the mandate period, psychiatric provision within Palestine expanded significantly, starting with the establishment of a government mental hospital at Bethlehem in 1922. A lack of alternative provision across the region had always been highlighted by the authorities at ʿAsfuriyyeh as a key reason it drew patients from near and far. The hospital had been ‘the only hospital for mental diseases in the whole Turkish empire between Constantinople and Cairo’, it was observed in 1927—that is, ‘until the arrival of the British in Palestine, when a small government hospital of seventy beds was started in Bethlehem’.^63^ Provision in Palestine continued to grow, with a second government mental hospital, again at Bethlehem, opening its doors in 1932, and a third, this time at Jaffa, established in 1944. There was also a dramatic expansion in private Jewish provision, as an increasing number of European Jewish psychiatrists came to Palestine, particularly from the 1930s, and founded private institutions in Jerusalem, Tel Aviv, and Haifa.^64^

Yet if psychiatric provision within Palestine grew, supply never kept up with demand, forcing some families to look elsewhere of necessity. After initially hailing the opening of the new mental hospital at Bethlehem, the authorities at ʿAsfuriyyeh quickly recognised this. In 1934, Dr Watson Smith remarked:

In Palestine… there is a small place for mental patients at Bethlehem. But that is completely full, and the accommodation is far from satisfactory. So also is the other place in Palestine, which has been run for many years by the Jews. The patients are absolutely packed together. These two comprise all the accommodation given for mental patients in Palestine. I visited the government offices, and was told that they had a list of 40 patients waiting for admission to these two crowded places.^65^

By the end of the decade, the situation had deteriorated further, such that the waiting list for a bed in a government mental hospital in Palestine had surpassed three hundred.^66^ But Watson Smith’s comment also draws attention to the question of standards, as well as supply. Mental hospitals in Palestine were not only overcrowded; they were also increasingly associated with criminality, as impossibly long waiting lists pushed desperate families to turn to criminal legal proceedings in order to secure institutionalisation for unwell relatives.^67^ Across the interwar decades, then, the hospital authorities continued to insist that for many, admission to ʿAsfuriyyeh was a choice rather than an option of last resort. Watson Smith shared an anecdote to this effect in 1927, about a man of ‘noble family’ from Mecca who had been brought to the hospital. When he asked the man’s relatives why they had not taken him to an institution at Bethlehem, or indeed Cairo, instead, ‘they told me that, after inquiry, they had come to the conclusion that this hospital was the best’. He added: ‘The patient got better.’^68^

Retracing the steps of a late Ottoman generation of psychiatric patients from Palestine to ʿAsfuriyyeh may also have been about more than just rational calculation. For some, the Lebanon Hospital for Mental Diseases may simply have continued to appear a more ‘local’ option than government institutions in Bethlehem. As in the period before the First World War, admissions statistics—available up until 1936—reveal that, leaving aside the two most populous cities in Palestine, Jerusalem and Jaffa-Tel Aviv, most patients continued to come to ʿAsfuriyyeh from towns across the north of Palestine: from Haifa in particular, but also Acre, Nazareth, Safad, and the Jewish colony of Rosh Pinah, and a little further to the south, Nablus, too. Towns like Haifa, Acre, and Nablus had orbited Beirut in the late Ottoman period; this gravitational pull appears to have had a long afterlife. The itineraries of psychiatric patients, then, as much as those of pilgrims, smugglers, and others, make clear that the new border did not completely sever ties between parts of the former Ottoman province of Beirut.^69^

It would be a mistake, however, to think about cross-border mobilities in the interwar Levant solely in terms of holdovers from older patterns that persisted in spite of the imposition of the mandates and their borders. Instead, as Toufoul Abou-Hodeib has argued, these patterns of mobility were dynamic, transformed by borders, and negotiated by states and subjects.^70^ This is as true of psychiatric mobilities as other kinds of mobilities; they did not endure entirely unchanged. In the first place, the proportion of Muslim and Jewish patients from Palestine at ʿAsfuriyyeh reversed in the interwar years. While Jewish patients made up nearly half of admissions to ʿAsfuriyyeh, and Muslim patients just a fifth, before the First World War, these proportions reversed completely thereafter. (The proportion of Christian patients stayed level, representing a third of admissions from Palestine.) The mandate period saw a profound reworking of Palestine’s social fabric, as the British committed themselves through the 1917 Balfour Declaration to the establishment of a Jewish national home in Palestine, and supported the Zionist movement’s efforts to build up their own autonomous parastatal institutions, including in the field of healthcare. Within Palestine at least, missionaries certainly perceived Zionist investment in parallel health and education systems as aimed at making ‘both Christian school and hospital superfluous so far as the Jew is concerned’.^71^ Given the proliferation of private Jewish psychiatric institutions in Palestine in the 1930s, it is not a stretch to extend this line of thinking to ʿAsfuriyyeh: it was increasingly surplus to requirements.

Another notable change was that opportunities for return migration were severely restricted as a result of legislative changes by the mandate government. As Lauren Banko has shown, the history of Palestinian return migration after the First World War was one of ever-increasing difficulty and constraint. This was the case already from 1918, when a large number of Arabs living abroad found themselves without valid passports and unable to enter Palestine as members of its indigenous population.^72^ The challenges only deepened over time: excluded from Palestinian citizenship by the 1925 Citizenship Order-in-Council, many Palestinians abroad had to take Turkish citizenship as former Ottoman subjects in order to have consular protection and travel to Palestine on a recognised passport.^73^ These difficulties were compounded for psychiatric cases among prospective return migrants by a further legal development in 1925: that year, the mandate government also enacted an ordinance restricting immigration on the grounds of medical unfitness, a restriction many governments around the world had introduced since the late nineteenth century.^74^ Targeted for exclusion was any foreigner who was ‘a lunatic, idiot, or mentally deficient’.^75^ In line with this, the mandate’s department of health kept track of those who were citizens in the government mental hospital in the 1920s, and those who were not, distinguishing them so they could be marked for deportation.^76^ If in the period before the First World War psychiatric mobilities sometimes entangled trans-Atlantic return migration and regional therapeutic itineraries, after 1925 the conditions of possibility for the first link in that chain—the return migration of mentally ill Palestinians—were severely curtailed.^77^ Histories of psychiatry have long charted the global spread of eugenicist immigration laws that aimed to identify and exclude the mentally ill from the late nineteenth century onwards.^78^ The case of Palestinian return migration, so important in the late Ottoman period, starkly illustrates how these laws might intersect with other exclusionary logics to extinguish pre-existing therapeutic itineraries.

Palestinians continued to travel to the Lebanon Hospital for Mental Diseases as Ottoman rule gave way to the British and French mandates. But even as these patterns of psychiatric mobility endured the transition between empires, they were stretched and warped by new developments, whether the instantiation of a colonial border, the uneven expansion of psychiatric provision within Palestine, or the introduction of increasingly exclusionary immigration regimes. But there are two further areas in which Abou-Hodeib’s call to attend to the dynamic nature of interwar mobilities can be taken up: how these patterns of psychiatric mobility were influenced by changing political circumstances, above all by the outbreak of anti-colonial revolt in the late 1930s; and how families made use of the new colonial border, transforming it from an obstacle to a resource in their strategies to achieve relief for—as well as perhaps from—mentally ill relatives. The final part of this article explores both areas in detail.

## Families, politics, and the border

Just as families played an important role in the admission and discharge, as well as institutional experiences, of Palestinian patients at ʿAsfuriyyeh before the First World War, so too were they key actors in shaping psychiatric mobilities in the mandate years. Far from mechanically reproducing earlier patterns of managing mental illness, families responded dynamically to the major shifts of this period. This is especially clear in the tumultuous decade or so which followed the outbreak of the great Palestinian revolt in 1936, a decade scarred by anti-colonial rebellion, a brutal colonial counterinsurgency, and the dislocations of the Second World War. Across these years, families responded to growing pressures within Palestine by looking north, and appear to have begun to turn the border to their advantage, playing mandate officials in Palestine off against hospital authorities in Lebanon. Rather than enduring in spite of the colonial partition of the region, some of the strategies examined here would seem to have been only made possible by the border.

Though protests against Zionism and British rule had punctuated the decade immediately following the First World War, 1936 saw the beginning of the most sustained and widespread uprising of the mandate period. Starting in April 1936 with a unprecedented months-long strike by Palestinians in the cities, by the end of the year resistance had morphed into a guerrilla-style insurgency in the countryside that was only quashed on the eve of the Second World War as a result of a British counter-insurgency effort involving, at its height, tens of thousands of troops.^79^ Historians of psychiatry have long been alive to how psychiatric expertise could be marshalled in moments of crisis to de-legitimise dissent and shore up colonial rule.^80^ But the case of the great revolt suggests another way of understanding the relationship between histories of politics and psychiatry, directing attention to how the management of mental illness within society might change in response to political circumstances.

That the management of mental illness did change is most bluntly suggested by admissions figures for the Lebanon Hospital for Mental Diseases. The number of patients admitted from Palestine most years hovered in the single digits, as we have seen. But between 1937 and 1939—precisely those years in which the great revolt and its suppression ravaged Palestinian society—the number of Palestinians who entered the hospital rose to sixty, a remarkable spike in admissions. Families did not, it seems, make decisions about how to manage mental illness in a vacuum set apart from politics. But what precisely drove this dramatic increase in the number looking to Beirut in the late 1930s?

In the first place, the great revolt had a grim impact on the workings of already-overburdened government mental institutions. The government hospital at Gaza was bombed in July 1938, and later that year, another incident exposed the vulnerability of mental hospitals specifically, when armed men entered the first government mental hospital in Bethlehem and murdered a Jewish member of the hospital staff.^81^ This shocking event led George Heron, the director of health, to request that two British police officers be billeted to guard the hospital at night, and that the doors and windows of the hospital be strengthened, with iron shutters and iron doors installed to secure the staff dormitories.^82^ Heron sought to minimise any disruption the posting of guards might entail.^83^ But against this backdrop, it would hardly be surprising if Palestinians came to see sending unwell relatives away as a safer option.^84^

It seems that the director of health’s attempt at business as usual, however, was largely mirrored by a continued demand for admission among Palestinians. Given the wider strain placed on relations between state and society in these years, it is remarkable that Palestinians continued, as in previous years, to address petitions to the mandate government requesting the institutionalisation of mentally ill relatives.^85^ In 1937, the department of health reported it was receiving ‘daily applications and petitions from relatives and friends for the admission of patients for whom there are no vacancies and whose presence in the home results in the complete disorganisation of all family and social life’. In line with this, the number awaiting admission had leapt to 246, up from 179 the previous year—a notable surge.^86^

Khader D. of Bayt Hanina was one of many, then, when he wrote to the department of health in January 1937 to request that his wife, Yamineh, be admitted to the ‘Bethlehem women hospital for fool-persons for treatment’.^87^ He was clearly desperate, for he escalated matters by writing to the High Commissioner—the top official within the mandate government—just a few days later with the same request, explaining how his wife had been ‘very dangerously and seriously insane’ for the past year and a half.^88^ But Yamineh, it seems, was not admitted to the government mental hospital. While families continued, in spite of the threat of violence that shadowed hospitals, to turn to the mandate government for aid, the case of Yamineh highlights the difficulties families faced in accessing government provision—difficulties that could lead them to look outside the borders of Palestine instead. Such was the increase in pressure on government provision, and the importance of ʿAsfuriyyeh in relieving some of that pressure, that in an extraordinary throwaway remark at a meeting of the Lebanon Hospital for Mental Diseases’ general committee in 1938, it was revealed that the mandate’s director of health had approached the hospital committee with the suggestion ‘that a home for private mental cases should be established by the committee on Mount Carmel’, in Haifa.^89^ Nothing ever came of this proposal.

The dramatic increase in the number of admissions to ʿAsfuriyyeh must be seen, then, in the context of a wider increase in the number of Palestinians seeking institutional treatment of one kind or other during the great revolt. This rise in demand for admission might reflect a number of developments. At least some cases admitted to ʿAsfuriyyeh in the late 1930s were causally linked to traumatic political violence by psychiatrists at the time.^90^ But as is suggested by the story of Yamineh, who remained at home for over a year before her husband approached the government, this rise in demand for admission might also be explained in terms of conditions during the great revolt making it impossible for families to continue caring for relatives—with sometimes long-standing issues—at home. The well-documented economic dislocations of these years represent one obvious factor at play here.^91^ Straitened finances will have had an ambiguous impact, however, on how families viewed the Lebanon Hospital for Mental Diseases, which was increasingly desperate for private patient fees by the late 1930s. Families who might have looked to government mental hospitals for relief when no longer able to cope with the cost of caring for mentally ill relatives at home would have had to think twice, when confronted by the fees charged by ʿAsfuriyyeh.

However difficult to pinpoint cause and effect, the tumultuous political circumstances of the late 1930s impacted on how families responded to mental illness, driving a sharp increase in admissions to the Lebanon Hospital for Mental Diseases. If families in this context appear reactive, buffeted by forces beyond their control, a closer look at the archive reveals families as more dynamic, more enterprising, perhaps even more ruthless, actors. They take on this appearance in a perhaps surprising context: in relation to their negotiation of the border between Palestine and Lebanon, which—after years of relative porosity—became dramatically harder in the late 1930s as the British stationed ever more police and soldiers, constructed police forts and a barbed-wire fence, and even mined the frontier as part of their counterinsurgency efforts.^92^ Yet the border was not just an obstacle to movement. It also, as Cyrus Schayegh has argued with respect to smuggling,^93^ created new opportunities. This becomes clear through careful examination of correspondence relating to the indebted families of Palestinian patients at the Lebanon Hospital for Mental Diseases in the early 1940s.

In the 1940s, the Lebanon Hospital for Mental Diseases was desperately seeking to recover debts owed by the families of private patients. By the late 1930s, the fall in the value of the franc and a sharp inflation in prices had put the hospital in serious financial difficulty, as the French mandate government and the Beirut municipality—together responsible for admitting and covering the fees of nearly a third of patients admitted in 1938^94^—refused to increase their contributions in line with inflation and indeed began withdrawing patients from the hospital to cut costs further.^95^ Under these enormous financial stresses, the hospital had little choice but to pursue the families of private patients for outstanding fees. While the medical superintendent did appeal directly to families living outside the borders of Lebanon, this was not always effective, and in desperation the hospital authorities turned to representatives of the relevant governments in Beirut for help. The mandate government of Palestine was not alone in being approached in this way: the hospital also wrote to the British Consul General of Iraq in the hope of securing assistance in the settlement of the debts of Iraqi patients, with no success.^96^

Though the British mandate government of Palestine was more willing to offer help, the scale of the problem of indebtedness among Palestinian patients was daunting—and the response by families complicated matters further. Two examples illustrate this. Tewfik A. had sent his wife, Anisa, to ʿAsfuriyyeh near the start of the outbreak of the great revolt. Described as a ‘well-to-do’ inhabitant of Jaffa by the hospital authorities, he was being charged £270 a year to meet his wife’s expenses as a first-class patient in the hospital—a princely sum.^97^ Yet by 1939, he had not paid the hospital what he owed for more than 2 years, a total of over £600. A second ‘well-to-do’ Jaffan, Dr Said D., had also failed to keep up with the costs of his brother’s treatment, and by 1939 owed the hospital nearly £300. The medical director of the hospital, Dr Stewart Miller, had sent a number of letters informing them that unless they cleared their debts, they would need to make arrangements to remove their relatives from ʿAsfuriyyeh. These letters had had no effect, and so the hospital turned to the mandate government for help. Miller asked that the two men be interviewed by a district commissioner so their intentions could be ascertained, and—if they were unable to continue paying—arrangements made for the removal of their relatives from the hospital.^98^ A district commissioner was duly dispatched to interview the pair; they informed him that they had each been able to arrange some reduction in their debts to the hospital. In the case of Tewfik A., he even claimed that he had been in Lebanon just a fortnight earlier, and had arranged the payment of the expenses with Dr Stewart Miller himself.^99^

This turned out to be a lie. Neither had come to any concrete arrangement with the hospital and Tewfik A. had not visited recently, as he claimed, but much earlier, and had never acted upon the promises he had made then. The district commissioner was dispatched once again to interview and ‘bring home to these two men of Jaffa a sense of their responsibility in the matter’.^100^ This time the district commissioner reported that Tewfik A. had left Jaffa to take up residence in Beirut at the Savoy Hotel, and that Dr Said D. was in the process of selling land in order to raise the necessary funds and would send a representative to Beirut shortly to reach an agreement with them.^101^ Revisited a fortnight later by the district commissioner, in the hope that sustained pressure would keep him to his word, Dr Said D. claimed that he had sent a cheque to the hospital as an interim measure.^102^ In April 1940, both were revealed once more to have lied: Dr Said D. had made no remittance to the hospital; more outrageously, the British consul in Beirut—acting as the intermediary between the hospital and the mandate government—reported that it had not ‘been possible to trace Mr Tewfik A. in Beirut; in fact his daughter informed the consulate this morning that her father was still living at… Jaffa’.^103^ In view of their repeated dishonesty, the consul advised the hospital that ‘nothing short of legal proceedings carried to a successful issue will compel them to discharge their obligations’.^104^ After a further round of exasperating interviews with both men, the High Commissioner agreed.^105^ The first letter to the mandate government about these two men had been sent in October 1939; it was May 1940 when this attempt at resolution outside the courts was finally abandoned.

These cases were not unique; indeed they were only the first in a series of cases that saw ʿAsfuriyyeh attempt to recover the debts owed by families in Palestine. They are worth relating in such detail, however, because they exemplify the creative ways in which Palestinians sought to exploit the circumstances that resulted from the admission of their relatives to an institution across the border in Lebanon. The need to have families arrange the transportation of their relatives back to Palestine before they could be discharged from ʿAsfuriyyeh; the delays in communication between mandate officials in Palestine, the British consul in Beirut, and the hospital’s director; and uncertainty about whether relatives were in Jaffa or Beirut at any given time, all gave Palestinians room for manoeuvre which they used to their advantage. To a degree, this room for manoeuvre shrank over time. While the mandate government had refused to repatriate Palestinian patients when their indebted relatives failed to do so, on the grounds that there were no vacancies available in government institutions in Palestine, the situation changed in 1944 with the opening of a new government mental hospital at Jaffa.^106^ With beds now available, forms were sent to ʿAsfuriyyeh to be completed for the transfer of patients to Palestine, and the families of five patients were informed that arrangements were being made to repatriate their relatives.^107^ Over summer 1945, four of the families scrambled to prevent this.^108^ Only in one instance did a husband admit he could no longer afford his wife’s maintenance; she was transferred to the Jaffa mental hospital at the beginning of October 1945.^109^

This episode, in which mandate officials found themselves chasing a number of families for the payment of their debts to the hospital, is revealing for a number of reasons. In the first place, while the decision to send relatives across the border to a private institution would seem to take these individuals and their families firmly beyond the control or concern of the mandate government, in reality these patients had the same potential to be foisted back on the mandate as a responsibility of government as did patients within the borders of Palestine when things went wrong. But second, this episode highlights how families could make strategic use of the new colonial border. Looking through the reports on these cases, it is striking how families utilised the delays in communication and uncertainties introduced by the Palestine-Lebanon border to their advantage, claiming to have sent fees, reached separate agreements with the hospital, and even themselves crossed the border, when they had not. Under the right circumstances, these manoeuvres enabled families to temporarily evade demands for repayment, and escape the constraints ordinarily placed on their use of private institutions by finances: Tewfik’s wife Anisa, for instance, remained at the Lebanon Hospital for Mental Diseases for years, in spite of his failure to pay for her upkeep, and ultimately, his debt to the hospital—totalling nearly £700—was written off as irrecoverable in 1948.^110^ The virtuosity of these ‘well-to-do’ families is in sharp contrast to the more usual portrayal of the Palestinian middle classes or elites as always ‘doggedly out of step with the times’.^111^

A third point follows. It is tempting to cast histories of interwar mobility as acts of defiance in the face of a crude colonial carve-up of a long-interconnected region, and to valorise the families examined here who played the border to their benefit. But that is only if colonialism is our singular point of reference for framing their actions, and the state the sole context of coercion we need to consider. Back in the 1980s, Mark Finnane drew attention to the agency of families in plotting the admission of mentally ill relatives to institutions, in a rejoinder to the notion of the ‘innocent’ family forced to give up the care of relatives by circumstances beyond their control.^112^ Historians and anthropologists have developed this in the decades since, none perhaps as starkly as João Biehl, in his ethnographic study of the social abandonment of the mentally ill and others in contemporary Brazil that results when ‘the family gets rid of its undesirable members’.^113^ That families could act coercively, especially towards female relatives, is evident in this context from the infamous cases of the psychiatric institutionalisation of the intellectual May Ziadeh in the 1930s and the actress Darina Al-Joundi decades later.^114^ Recovering such family dynamics from the archival material examined here is far from straightforward. We are left to speculate as to whether those rich Jaffan men who disposed of wives and brothers at ʿAsfuriyyeh, and refused to either pay for their treatment or make arrangements to bring them home, did so because they thought the continuation by any means necessary of their stay at ʿAsfuriyyeh was in the best interests of their health—or because they judged ʿAsfuriyyeh to be a convenient place to rid themselves of these inconvenient people.^115^ Was it the unparalleled standard of care on offer at the hospital, or the fact that it lay over the border where relatives could be put more easily out of sight and out of mind, that appealed more?

The hospital’s own view on the matter was clear. Long before these debt cases appeared on the horizon, the hospital’s medical director lamented:

It is an unfortunate but none the less remarkable fact that when once an insane person has been ‘put away’ it is astonishing how quickly he is forgotten. For destitute patients one expects nothing, and not much more from those who have been admitted through the instrumentality of missionaries and other charitable workers, but it is extraordinary how dilatory and casual many relatives become, even of the ordinary creature comforts of those belonging to them.^116^

In a sense, the intentions of families were unimportant. As Patricia Prestwich writes, in a very different context, the best of intentions will have done little to mitigate any ‘feelings of imprisonment, isolation, and abandonment that patients experienced’.^117^

## Conclusion

In the late 1940s, Adma K.—a stenographer from Jerusalem who had worked for more than a decade for various government departments, as well as the army during the Second World War—was admitted to the Lebanon Hospital for Mental Diseases. In February 1947, she had fallen ill with symptoms of agitation and marked persecutory delusions, according to her brother, and had been admitted to the Bethlehem mental hospital, where she received ten courses of electro-convulsive therapy. Discharged as apparently recovered, Adma quickly suffered a recurrence of her symptoms and became difficult to manage at home; this time she was admitted to ʿAsfuriyyeh. She recalled her admission vividly: she told the examining doctors at the hospital that she had been ‘kidnapped by her brother and a motor car driver and thrown into ʿAsfuriyyeh’, and compared herself to May Ziadeh, who ‘was also misjudged and placed here’.^118^ Her case file bulges with correspondence—handwritten and typed—pleading with family members, hospital authorities, and even the State of Israel to secure her release. Given a diagnosis of chronic schizophrenia, she was still, however, a patient in the hospital well into the 1960s, when her file peters out.

The atypical fullness and survival of her records notwithstanding, in outline Adma’s story echoes many of those at the heart of this article, of Palestinian patients who were brought to ʿAsfuriyyeh for treatment across the first half of the twentieth century. But it also represents a fundamental rupture with them, because it was not the prospect of psychiatric treatment that propelled her north. Instead, Adma was one of hundreds of thousands of Palestinians displaced between 1947 and 1948, when the newly created United Nations General Assembly voted to partition Palestine, the British mandate came to an end, and the State of Israel was established. By the end of 1948, more than half of the Palestinian population—around 750,000 people—had been uprooted, over five hundred villages destroyed, and 80% of the territory of mandate Palestine incorporated into the new State of Israel.^119^ Adma was one of many who had come north during the ‘exodus of Palestinians’, as her examining doctor put it.^120^ If the instantiation of a border between the British and French mandates had transformed longstanding patterns of travel northwards for treatment from regional into transnational movement, the Palestinian *nakba* or catastrophe of 1948 led to further resignifications, as those who found their way to the Lebanon Hospital for Mental Diseases might now be counted under a new category with a staying power few at the time could have predicted: ‘Palestinian refugee’.^121^ With 1948 witness to the highest number of admissions from Palestine in the history of the hospital to date, the great historian Albert Hourani—speaking at the annual meeting of the hospital’s general committee in London the following year—put events in context.^122^ During the hospital’s lifetime, he reminded his audience, Lebanon had been called upon to respond twice to major regional calamities: the Armenian genocide three decades earlier, which had resulted in thousands of Armenians settling in Beirut; and now, Hourani warned, ‘the same thing is happening with the Arab refugees from Palestine’.^123^

When the Lebanon Hospital for Mental Diseases first opened its doors in August 1900, it entered into and drew life from a rich, complex world of motion, a world which warped and bent but nonetheless endured across decades of profound political and social dislocations. Focussing on the case of Palestinian patients at the hospital reveals how these seismic shifts might be weathered by families reckoning with challenges on a far more intimate scale, and underlines the need to escape the stubbornly national framing of much of the scholarship on colonial psychiatry by adopting a transnational perspective and embracing multi-sited archival research. Returning to the story of Tewfik and Anisa yields a poignant, final testament to this; it is a story that cannot be made sense of within the confines of post-Ottoman borders. When the hospital’s efforts to negotiate a repayment of outstanding fees with Tewfik failed, and arrangements were being made to repatriate Anisa to Palestine, it emerged that Anisa held a Sudanese rather than Palestinian passport, issued at Khartoum in 1935. Complicating matters further, her passport showed her place of birth as Latakia in Syria, not Jaffa, as the hospital had believed.^124^ Anisa and her husband’s story, taking in Khartoum, Latakia, Jaffa, and Beirut, was not anomalous; as Sherene Seikaly has stressed, this was the world of circulation and mobility inhabited by well-heeled Palestinians in the period before 1948.^125^ For the inhabitants of this world of movement, why would responses to mental illness be any less agile, any less peripatetic? But this world was not endlessly resilient, and the coda to this story—the writing off of Tewfik’s debt to the hospital amidst the catastrophe of 1948—might well be taken as marking an end point of sorts for the pattern of psychiatric mobilities that had defined the first half of the twentieth century: having weathered the storms of war and the passage of empires, that world was to change forever with the Palestinian *nakba* of mass forced displacement in 1948.

## Footnotes

1 For the early history of the hospital, see Joelle Abi-Rached, ʿ*Aṣfūriyyeh: A History of Madness, Modernity, and War in the Middle East* (Cambridge: MIT Press, 2020), 49–76.

2 Third Annual Report, Lebanon Hospital for the Insane [LHMD], 1901, 14–15. All annual reports are from the Saab Medical Library, American University of Beirut [AUB].

3 Ibid., 16.

4 The name came from the area in Mount Lebanon where the hospital had been established, though its meaning in Arabic—the place of the birds—evokes an enduring and stigmatising representation of the psychiatric hospital as a kind of bird-cage. Like the names of psychiatric institutions the world over (‘Bedlam’), the name took on a life of its own and the hospital authorities ultimately sought to distance themselves from it. See Abi-Rached, ʿ*Aṣfūriyyeh,* 2, 203, 94.

5 These figures derive from the hospital’s annual reports, which recorded the place of residence of patients first by city (e.g., Jerusalem, Jaffa) and then from the mid-1930s by nationality (e.g., Palestine).

6 Chris Sandal-Wilson, *Mandatory Madness: Colonial Psychiatry and British Mandate Palestine* (Cambridge: Cambridge University Press, 2023), 37–41.

7 Ibid.

8 For South-South therapeutic itineraries, see: Beth Kangas, ‘Therapeutic Itineraries in a Global World: Yemenis and their Search for Biomedical Treatment Abroad’, *Medical Anthropology,* 21 (2002), 35–78; Meghann Ormond and Dian Sulianti, ‘More Than Medical Tourism: Lessons from Indonesia and Malaysia on South-South Intra-Regional Medical Travel’, *Current Issues in Tourism,* 20 (2017), 94–110; Omar Dewachi et al., ‘(Dis)connectivities in Wartime: The Therapeutic Geographies of Iraqi Healthcare-seeking in Lebanon’, *Global Public Health,* 13 (2018), 288–97.

9 Toufoul Abou-Hodeib, ‘Involuntary History: Writing Levantines into the Nation’, *Contemporary Levant,* 5 (2020), 50.

10 This is particularly important in histories of colonial psychiatry, which have tended to produce rich case studies of individual institutions and colonial contexts. For notable exceptions, see Matthew Heaton, ‘Aliens in the Asylum: Immigration and Madness in Gold Coast’, *Journal of African History,* 54 (2013), 373–91; Matthew Heaton, ‘Contingencies of Colonial Psychiatry: Migration, Mental Illness, and the Repatriation of Nigerian “Lunatics”’, *Social History of Medicine,* 27 (2013), 41–63; Claire Edington and Hans Pols, ‘Building Psychiatric Expertise Across Southeast Asia: Study Trips, Site Visits, and Therapeutic Labour in French Indochina and the Dutch East Indies, 1898–1937’, *Comparative Studies in Society and History*, 58 (2016), 636–63. For the postcolonial period, see Richard Keller, *Colonial Madness: Psychiatry in French North Africa* (Chicago: University of Chicago Press, 2007), 191–226.

11 Abigail Jacobson, *From Empire to Empire: Jerusalem Between Ottoman and British Rule* (Syracuse: Syracuse University Press, 2011); Jacob Norris, *Land of Progress: Palestine in the Age of Colonial Development, 1905–1948* (Oxford: Oxford University Press, 2013); Cyrus Schayegh and Andrew Arsan, eds, *The Routledge Handbook of the History of the Middle East Mandates* (Abingdon: Routledge, 2015); Michael Provence, *The Last Ottoman Generation and the Making of the Modern Middle East* (Cambridge: Cambridge University Press, 2017).

12 Abou-Hodeib, ‘Involuntary History’, 44–53. See also Cyrus Schayegh, ‘The Many Worlds of ‘Abduh Yasin: or, What Narcotics Trafficking in the Interwar Middle East Can Tell Us about Territorialisation’, *American Historical Review,* 116 (2011), 273–306; Toufoul Abou-Hodeib, ‘Sanctity Across the Border: Pilgrimage Routes and State Control in Mandate Lebanon and Palestine’, in Cyrus Schayegh and Andrew Arsan, eds, *The Routledge Handbook of the History of the Middle East Mandates* (Abingdon: Routledge, 2015), 383–94; Haggai Ram, ‘Hashish Traffickers, Hashish Consumers, and Colonial Knowledge in Mandatory Palestine’, *Middle Eastern Studies,* 52 (2016), 546–63.

13 Abi-Rached, *ʿAsfuriyyeh,* 20.

14 For the role of the family in histories of psychiatry, see for instance: David Wright, ‘Getting out of the Asylum: Understanding the Confinement of the Insane in the Nineteenth Century’, *Social History of Medicine,* 10 (1997), 137–55.

15 To paraphrase Claire Edington, *Beyond the Asylum: Mental Illness in French Colonial Vietnam* (Ithaca: Cornell University Press, 2019), 6–7.

16 List of Patients at the Lebanon Hospital for the Insane, 1 August 1900 to 31 March 1936, Archives and Special Collections, Jafet Library, AUB.

17 Tenth Annual Report, LHMD, 1908, 29.

18 Heaton, ‘Aliens in the Asylum’, 380–81. Historians of psychiatry have given more attention to European migrants, especially the Irish. See: Angela McCarthy and Catharine Coleborne, eds, *Migration, Ethnicity, and Mental Health: International Perspectives, 1840–2010* (Abingdon: Routledge, 2012); Pauline Prior, ed, *Asylums, Mental Health Care and the Irish, 1800–2010* (Newbridge: Irish Academic Press, 2012); Catherine Cox et al., ‘Emaciated, Exhausted and Excited: The Bodies and Minds of the Irish in Nineteenth-Century Lancashire Asylums’, *Journal of Social History,* 46 (2012), 500–24; Catherine Cox and Hilary Marland, ‘“A Burden on the County”: Madness, Institutions of Confinement and the Irish Patient in Victorian Lancashire’, *Social History of Medicine,* 28 (2015), 263–87.

19 This is not to deny the salience of Palestinian identity prior to the First World War: see Rashid Khalidi, *Palestinian Identity: The Construction of Modern National Consciousness* (New York: Columbia University Press, 1997).

20 Eighth Annual Report, LHMD, 1906, 19–21.

21 Abi-Rached, *ʿAsfuriyyeh,* 103, 127.

22 R. M. Ion and M. D. Beer, ‘The British reaction to dementia praecox, 1893–1913’, *History of Psychiatry,* 13 (2002), 285–304.

23 Gershon Shafir, *Land, Labor and the Origins of the Israeli-Palestinian Conflict, 1882–1914* (Oakland: University of California Press, 1996), 45–90.

24 Entry 88, LHMD Case Book (Male), 1 November 1904–13 December 1909, Archives and Special Collections, Jafet Library, AUB. Throughout this article to protect patient anonymity only first names and an initial are used for patients and close relatives.

25 Abi-Rached, *ʿAsfuriyyeh,* 107–108. For the limits of this explanation, see Beverly A. Tsacoyianis, *Disturbing Spirits: Mental Illness, Trauma, and Treatment in Modern Syria and Lebanon* (Notre Dame: University of Notre Dame Press, 2021), 66.

26 Second Annual Report, LHMD, 1900, 8.

27 Eighth Annual Report, LHMD, 1906, 19–21.

28 Ellen Fleischmann, ‘The Impact of American Protestant Missions in Lebanon on the Construction of Female Identity, c. 1860–1950’, *Islam and Muslim-Christian Relations,* 13 (2002), 411–26; Ruth Kark, ‘The Impact of Early Missionary Enterprises on Landscape and Identify Formation in Palestine, 1820–1914’, *Islam and Christian-Muslim Relations,* 15 (2004), 209–35; Liora Halperin, ‘The Battle over Jewish Students in the Christian Missionary Schools of Mandate Palestine’, *Middle Eastern Studies,* 50 (2014), 737–54; Ussama Makdisi, *The Artillery of Heaven: American Missionaries and the Failed Conversion of the Middle East* (Ithaca: Cornell University Press, 2017); Michael Marten, ‘Scottish Presbyterian Churches and Humanitarianism in the Interwar Middle East’, in Inger Marie Okkenhaug and Karène Sanchez-Summerer, eds, *Christian Missions and Humanitarianism in the Modern Middle East, 1850–1950: Ideologies, Rhetoric, and Practices* (Leiden: Brill, 2020), 209–30.

29 For ‘relational’ approaches to the history of mandate Palestine, see Zachary Lockman, ‘Railway Workers and Relational History: Arabs and Jews in British-Ruled Palestine’, *Comparative Studies in Society and History*, 35 (1993), 601–27. For the late Ottoman period, see Michelle U. Campos, *Ottoman Brothers: Muslims, Christians, and Jews in Early Twentieth-Century Palestine* (Stanford, CA: Stanford University Press, 2011).

30 Chris Sandal-Wilson, ‘Ethnographies of madness: Père Antonin Jaussen, Shaykh Sa’ad al-Din, and the management of mental illness in mandate-era Nablus’, in Sarah Irving, ed, *The Social and Cultural History of Palestine: Essays in Honour of Salim Tamari* (Edinburgh: Edinburgh University Press, 2023), 142–72; Sandal-Wilson, *Mandatory Madness,* 40–47.

31 Ezrath Nashim Information Booklet, c.1929, Israel State Archives [ISA] P 4270/11, 19.

32 Ibid., 19.

33 Bulletin No. 20, for 1922, of the Home for Insane and Incurable founded by the Ezrath Nashim Society, Jerusalem, ISA M 6627/23.

34 Ibid. See also Sandal-Wilson, *Mandatory Madness,* 49–50.

35 Abi-Rached, *ʿAsfuriyyeh*, 134–35.

36 Tel Aviv, founded in 1909, was not listed separately.

37 Beshara Doumani, *Rediscovering Palestine: Merchants and Peasants in Jabal Nablus, 1700–1900* (Berkeley: University of California Press, 1995), 68–73; Jens Hanssen, *Fin De Siècle Beirut: The Making of an Ottoman Provincial Capital* (Oxford: Oxford University Press, 2005), 60; 166–80.

38 Abi-Rached, *ʿAsfuriyyeh*, 105–106.

39 Akram Khater, *Inventing Home: Emigration, Gender, and the Middle Class in Lebanon, 1870–1920* (Berkeley: University of California Press, 2001), 48.

40 Third Annual Report, LHMD, 1901, 16.

41 Ninth Annual Report, LHMD, 1907, 42.

42 Catherine Cox and Hilary Marland, ‘Introduction’, in Catherine Cox and Hilary Marland, eds, *Migration, Health and Ethnicity in the Modern World* (Basingstoke: Palgrave Macmillan, 2013), 8.

43 They have begun to receive increased scholarly attention, too: Nadim Bawalsa, *Transnational Palestine: Migration and the Right of Return before 1948* (Stanford: Stanford University Press, 2022); Jacob Norris, *The Lives and Deaths of Jubrail Dabdoub: Or, How the Bethlehemites Discovered Amerka* (Stanford: Stanford University Press, 2023).

44 Jacob Norris, ‘Return Migration and the Rise of the Palestinian Nouveaux Riches, 1870–1925’, *Journal of Palestine Studies,* 46 (2017), 65–66; 72.

45 Ibid. 62.

46 Entry 622, Case Book (Male), AUB.

47 For examples, see Entry 554 and Entry 718, Case Book (Male), AUB.

48 See Abi-Rached, *ʿAsfuriyyeh*, 99–135; Lamia Moghnieh, ‘The Broken Promise of Institutional Psychiatry: Sexuality, Women and Mental Illness in 1950s Lebanon’, *Culture, Medicine, and Psychiatry,* 47 (2023), 82–98.

49 Entry 726, Case Book (Male), AUB.

50 Entry 599, Case Book (Male), AUB.

51 Entry 609, Case Book (Male), AUB.

52 Entries 809 and 1082, Case Book (Male), AUB.

53 Abi-Rached, *ʿAsfuriyyeh*, 68–69.

54 Leila Tarazi Fawaz, *A Land of Aching Hearts: The Middle East in the Great War* (Cambridge: Harvard University Press, 2014), 83–105; Salim Tamari, *Year of the Locust: A Soldier’s Diary and the Erasure of Palestine’s Ottoman Past* (Oakland: University of California Press, 2015).

55 Seventeenth Annual Report, LHMD, 1915–16, 2.

56 Nineteenth Annual Report, LHMD, 1917–18, 1.

57 Lauren Banko, ‘Border Transgressions, Border Controls: Mobility along Palestine’s Northern Frontier, 1930–1946’, in Jordi Tejel and Ramazan Hakkı Öztan, eds, *Regimes of Mobility: Borders and State Formation in the Middle East, 1918–1946* (Edinburgh: Edinburgh University Press, 2022), 263–65.

58 Ibid., 272.

59 Twenty-First Annual Report, LHMD, 1919–20, 2.

60 Twenty-Fifth Annual Report, LHMD, 1923–24, 27.

61 Thirty-Sixth Annual Report, LHMD, 1934–35, 25.

62 See above, in particular Schayegh and Arsan, eds, *Routledge Handbook of the History of the Middle East Mandates.*

63 Twenty-Eighth Annual Report, LHMD, 1926–27, 23.

64 Sandal-Wilson, *Mandatory Madness,* 233–53; Rakefet Zalashik, *Das Unselige Erbe: Die Geschichte der Psychiatrie in Palästina und Israel* (Frankfurt am Main: Campus Verlag, 2012), 55–67.

65 Thirty-Fifth Annual Report, LHMD, 1933–34, 18.

66 Annual Report, Department of Health of the Government of Palestine, 1939, 73.

67 Sandal-Wilson, *Mandatory Madness,* 194–230.

68 Twenty-Seventh Annual Report, LHMD, 1926–27, 25.

69 Abou-Hodeib, ‘Involuntary History’, 45.

70 Ibid., 51.

71 Marten, ‘Scottish Presbyterian Churches and Humanitarianism’, 222.

72 Lauren Banko, *The Invention of Palestinian Citizenship, 1918–1947* (Edinburgh: Edinburgh University Press, 2016), 88.

73 Ibid., 110.

74 Sandy Sufian, ‘Mental Hygiene and Disability in the Zionist Project’, *Disability Studies Quarterly,* 27 (2007), n.p.

75 Acting Director of Immigration to Chief Secretary, Jerusalem, 6 February 1947, ISA M 4342/42.

76 Lauren Banko, ‘Keeping Out the “Undesirable Elements”: The Treatment of Communists, Transients, Criminals, and the Ill in Mandate Palestine’, *Journal of Imperial and Commonwealth History,* 46 (2019), 1169–70.

77 For an example, see Banko, *Invention of Palestinian Citizenship,* 185.

78 Ian Dowbiggin, *Keeping America Sane: Psychiatry and Eugenics in the United States and Canada, 1880–1940* (Ithaca: Cornell University Press, 1997); Alison Bashford, ‘Insanity and Immigration Restriction’, in Angela McCarthy and Catharine Coleborne, eds, *Migration, Ethnicity, and Mental Health: International Perspectives, 1840–2010* (Abingdon: Routledge, 2012), 14–35.

79 For a recent account, see Matthew Kelly, *The Crime of Nationalism: Britain, Palestine, and Nation-Building on the Fringe of Empire* (Berkeley: University of California Press, 2017).

80 Jock McCulloch, *Colonial Psychiatry and ‘The African Mind’* (Cambridge: Cambridge University Press, 1995); Hilary Sapire and Robert Edgar, *African Apocalypse: The Story of Nontetha Nkwenkwe, a Twentieth-Century South Africa Prophet* (Athens: Ohio University Press, 1999); Sloan Mahone, ‘The Psychology of Rebellion: Colonial Medical Responses to Dissent in British East Africa’, *Journal of African History,* 47 (2006), 241–58.

81 See ISA M 6599/3.

82 Director of Medical Services to District Commissioner, Jerusalem, 20 December 1938, ISA M 4087/9.

83 Ibid.

84 How this affected Jewish patients in particular is difficult to determine, because the records that would allow us to track the proportion of patients at ʿAsfuriyyeh from Palestine from different religious backgrounds are not extant for the period after 1936. But private Jewish institutions within Palestine would have given Jewish families who no longer felt able to entrust their relatives to government hospitals alternatives closer at hand than Beirut.

85 Chris Wilson, ‘Petitions and Pathways to the Asylum in British Mandate Palestine, 1920–1948’, *Historical Journal,* 62 (2019), 451–71.

86 Annual Report, Department of Health of the Government of Palestine, 1937, 63.

87 Khader D., Bayt Hanina, to Inspector General, Public Health, 16 January 1937, ISA M 6627/28.

88 Khader D., Bayt Hanina, to High Commissioner, 23 January 1937, ISA M 6627/28.

89 Minutes of the General Committee of the Lebanon Hospital for Mental Diseases, London, 19 November 1938, LHMD 2/3, School of Oriental and African Studies.

90 Sandal-Wilson, *Mandatory Madness,* 142.

91 Matthew Hughes, *Britain’s Pacification of Palestine: The British Army, the Colonial State, and the Arab Revolt, 1936–1939* (Cambridge: Cambridge University Press, 2019), 82–94.

92 Schayegh, *The Middle East*, 262–63; Steven Wagner, *Statecraft by Stealth: Secret Intelligence and British Rule in Palestine* (Ithaca: Cornell University Press, 2019), 205–11; Hughes, *Britain’s Pacification of Palestine*, 173–75.

93 Schayegh, ‘The many worlds of ‘Abud Yasin’, 283.

94 Fortieth Annual Report, LHMD, 1938, 8.

95 Thirty-Ninth Annual Report, LHMD, 1937, 20–21; Minutes of the Executive Committee, 10 November 1939, LHMD Box 30, AUB.

96 Consul General of Iraq to Medical Director, Lebanon Hospital for Mental Diseases, 15 February 1939, LHMD C3D2B9, AUB.

97 British Consul, Beirut, to High Commissioner for Palestine, 31 October 1939, ISA M 551/19. For context, the average gross income of a fellah in 1930 was £40; after taxes and costs, this fell to £9–11. Mahmoud Yazbak, ‘From Poverty to Revolt: Economic Factors in the Outbreak of the 1936 Rebellion in Palestine’, *Middle Eastern Studies,* 36 (2000), 93–113.

98 British Consul, Beirut, to High Commissioner for Palestine, 31 October 1939, ISA M 551/19.

99 District Commissioner, Lydda, to Chief Secretary, 12 December 1939, ISA M 551/19.

100 British Consul, Beirut, to High Commissioner for Palestine, 7 January 1940, ISA M 551/19.

101 District Commissioner, Lydda, to Chief Secretary, 2 February 1940, ISA M 551/19.

102 District Commissioner, Lydda, to Chief Secretary, 14 March 1940, ISA M 551/19.

103 The hospital had a deep and long-standing relationship with British consular authorities in Beirut, with the British consul general recognised as ‘the protector of ʿAsfuriyyeh’ in its earliest years while under Ottoman rule. See Abi-Rached, *ʿAsfuriyyeh*, 61.

104 British Consul, Beirut, to High Commissioner for Palestine, 3 April 1940, ISA M 551/19.

105 High Commissioner for Palestine to British Consul, Beirut, 16 May 1940, ISA M 551/19.

106 Acting Chief Secretary, Jerusalem, to British Consul, Beirut, 14 June 1943, ISA M 551/19.

107 Chief Secretary, Jerusalem, to British Consul, Beirut, 13 February 1945, ISA M 551/19.

108 Director of Medical Services to Chief Secretary, 19 July 1945, ISA M 551/19.

109 Director of Medical Services to Acting Senior Medical Officer, Jaffa, 21 September 1945, ISA M 551/19.

110 Annual Report of the Director, 1948, LHMD C3D2B9, AUB, 4–5.

111 Sherene Seikaly, *Men of Capital: Scarcity and Economy in Mandate Palestine* (Stanford: Stanford University Press, 2015), 10.

112 Mark Finnane, ‘Asylums, Families, and the State’, *History Workshop,* 20 (1985), 137.

113 João Biehl, *Vita: Life in a Zone of Social Abandonment* (Berkeley: University of California Press, 2005), 14. Historians have been more ambivalent about family motives: Wright, ‘Getting Out of the Asylum’; Patricia Prestwich, ‘Family Strategies and Medical Power: “Voluntary” Committal in a Parisian Asylum, 1876–1914’, in Roy Porter and David Wright, eds, *The Confinement of the Insane: International Perspectives, 1800–1965* (Cambridge: Cambridge University Press, 2003), 79–99; Catharine Coleborne, ‘Families, Insanity, and the Psychiatric Institution in Australia and New Zealand, 1860–1914’, *Health and History,* 11 (2009), 65–82.

114 For discussion of another important, though less publicised, case, see Moghnieh, ‘The Broken Promise of Institutional Psychiatry’. See also Abi-Rached, *ʿAsfuriyyeh*, 128.

115 The phrase is from Andrew Scull, ‘A convenient place to get rid of inconvenient people: the Victorian lunatic asylum’, in Anthony D. King, ed, *Buildings and Society: Essays on the Social Development of the Built Environment* (London: Routledge, 1980), 37–60.

116 Thirty-Third Annual Report, LHMD, 1931–32, 19–20.

117 Prestwich, ‘Family Strategies and Medical Power’, 99.

118 Adma K., Reg. No. 9493, Interview with Patient and Medical Director Note, 19 August 1950, LHMD Box 2 and 3, AUB.

119 James Gelvin, *The Israel-Palestine Conflict: A History* (Cambridge: Cambridge University Press, 2021), 128, 138–39.

120 Adma K., Reg. No. 9493, Interview with Patient and Medical Director Note, 19 August 1950, LHMD Box 2 and 3, AUB.

121 Fiftieth Annual Report, LHMD, 1948, 4.

122 Ibid., 5. See also Abi-Rached, *ʿAsfuriyyeh,* 114.

123 Fiftieth Annual Report, LHMD, 1948, 13.

124 Consul General, Beirut, to Chief Secretary, Jerusalem, 10 March 1945, ISA M 551/19.

125 Sherene Seikaly, ‘How I Met My Great-Grandfather: Archives and the Writing of History’, *Comparative Studies of South Asia, Africa, and the Middle East*, 38 (2018), 8.

